# Repeated Relapse–Retreatment Cycles After Omalizumab Withdrawal in Chronic Spontaneous Urticaria: A Real-World Study

**DOI:** 10.3390/jcm15197657

**Published:** 2026-10-02

**Authors:** Hatice Celik Tuglu, Kurtuluş Aksu

**Affiliations:** 1 Department of Immunology and Allergy, Ankara Atatürk Sanatoryum Training and Research Hospital, Ankara 06290, Türkiye; kurtulusaksu@yahoo.com; 2 Department of Immunology and Allergy, Gebze Fatih State Hospital, Gebze, Kocaeli 41400, Türkiye

**Keywords:** chronic spontaneous urticaria, omalizumab, withdrawal, relapse, retreatment, step-down treatment

## Abstract

**Background/Objectives:** Relapse after omalizumab withdrawal is common in chronic spontaneous urticaria (CSU), but patients requiring repeated treatment re-initiation across successive withdrawal cycles remain poorly characterized. This study aimed to characterize patients with repeated relapse requiring omalizumab re-initiation and to determine whether routinely available baseline parameters could distinguish them from other patients with CSU. **Methods:** This retrospective, single-center study included 46 adults with CSU who achieved disease control with standard-dose omalizumab after an initial 6-month course. Patients were evaluated during a fixed 12-month period following the first withdrawal. Repeated omalizumab re-initiation was defined as relapse requiring re-initiation within 12 weeks after withdrawal on at least two occasions. Baseline clinical and laboratory characteristics were compared between groups, and multivariable logistic regression was performed. **Results:** Twenty-nine patients (63.0%) met the criteria for repeated omalizumab re-initiation. Baseline disease activity, angioedema, atopic status, autoimmune thyroid disease, previous systemic corticosteroid use, total immunoglobulin E (IgE), anti-thyroid peroxidase antibody (anti-TPO), C-reactive protein (CRP), eosinophil and basophil counts, and inflammatory indices did not differ significantly between the repeated-treatment and comparison groups. Vitamin B12 was the only laboratory parameter showing a significant between-group difference in univariable analysis (259 vs. 335 pg/mL; *p* = 0.034); however, this association was not retained after multivariable adjustment. None of the variables included in the multivariable model were independently associated with repeated omalizumab re-initiation. **Conclusions:** Repeated relapse requiring omalizumab re-initiation across successive withdrawal cycles was common during the 12-month observation period. Routinely available baseline clinical and laboratory parameters did not reliably distinguish patients who developed this relapse–retreatment pattern. These findings highlight the difficulty of predicting repeated retreatment requirements from conventional baseline assessments and support further prospective investigation of individualized withdrawal and step-down strategies in patients who develop recurrent relapse–retreatment cycles.

## 1. Introduction

Chronic spontaneous urticaria (CSU) is a mast cell-driven disorder in which wheals, angioedema, or both recur for longer than six weeks without an identifiable external trigger. Omalizumab is an effective and guideline-recommended add-on treatment for patients whose disease remains uncontrolled despite second-generation H1-antihistamines. Current management aims to achieve complete disease control while using as much treatment as necessary and as little as possible, with treatment step-down considered once sustained control has been achieved [1,2].

An important challenge in the long-term management of CSU is disease recurrence after omalizumab withdrawal. Real-world evidence indicates that relapse occurs in a considerable proportion of patients after withdrawal, while disease control can generally be restored when omalizumab is re-initiated [3,4]. Previous studies have mainly examined the frequency and timing of relapse after withdrawal and factors associated with recurrence [3,4,5]. More recently, a history of post-withdrawal relapse was independently associated with the risk of subsequent relapse, providing further rationale for evaluating outcomes across successive treatment-withdrawal cycles [5].

This distinction may have important implications for long-term treatment planning. In clinical practice, relapse after withdrawal may lead to omalizumab re-initiation and subsequent withdrawal attempts [2,4]. Individualized step-down strategies, including extension of dosing intervals, have been proposed to reduce treatment burden while maintaining disease control [2,4]. However, patients requiring repeated re-initiation shortly after successive withdrawals remain poorly characterized.

Therefore, this study aimed to characterize patients with CSU who experienced repeated relapse requiring omalizumab re-initiation within 12 weeks after withdrawal and to assess whether routinely available baseline parameters could distinguish these patients from those who did not develop this repeated relapse–retreatment pattern.

## 2. Materials and Methods

### 2.1. Study Design and Population

This retrospective, single-center study included adult patients with chronic spontaneous urticaria (CSU) who received omalizumab between 1 October 2014 and 30 October 2025. Clinical, laboratory, and treatment-related information was obtained retrospectively from electronic hospital records and patient files.

Eligibility criteria comprised age ≥18 years, achievement of disease control with standard-dose omalizumab (300 mg every 4 weeks), 12 months of documented clinical follow-up after the first treatment withdrawal, and availability of sufficient clinical and laboratory data. Disease control was determined retrospectively from the treating physician’s clinical assessment documented in the medical records, supported by available UAS7 and UCT assessments. Patients with insufficient clinical data or inadequate disease control with standard-dose omalizumab were excluded. After completion of the initial 6-month omalizumab course, clinical records from the 12-month period following the first treatment withdrawal were reviewed to determine the occurrence of relapse and the need for omalizumab re-initiation.

### 2.2. Omalizumab Treatment Protocol

All patients received omalizumab 300 mg every 4 weeks in accordance with international guideline recommendations [1]. Treatment withdrawal was considered after disease control had been achieved [2]. Omalizumab was re-initiated when recurrent CSU symptoms could not be adequately controlled with high-dose second-generation H1-antihistamines [1]. Given the retrospective nature of the study, decisions to withdraw or restart omalizumab were made in routine clinical practice and were not governed by a predefined study protocol.

### 2.3. Definition of Repeated Omalizumab Re-Initiation

Patients were considered to have repeated omalizumab re-initiation when treatment had to be restarted for relapse within 12 weeks of withdrawal in two or more treatment-withdrawal cycles during the 12-month observation period. A 12-week cut-off was chosen for this operational definition based on previous reports indicating that many post-withdrawal relapses occur during the first 3 months after omalizumab cessation [6,7,8,9].

In this study, a relapse qualifying for the outcome was considered to have occurred when recurrent CSU symptoms remained inadequately controlled despite high-dose second-generation H1-antihistamines and led to omalizumab re-initiation within 12 weeks of withdrawal. To differentiate this pattern from a single post-withdrawal relapse followed by one retreatment course, assignment to the repeated-treatment group required at least two qualifying relapse–retreatment cycles within the 12 months after completion of the initial 6-month omalizumab course, corresponding to at least two additional 3-month treatment courses. Patients meeting these criteria constituted the repeated-treatment group. Patients who fulfilled the eligibility criteria but did not meet the criterion for repeated omalizumab re-initiation during the same 12-month observation period constituted the comparison group. The comparison group was therefore defined by the absence of the prespecified repeated relapse–retreatment outcome and could include patients with different clinical courses after omalizumab withdrawal. The available retrospective records did not permit reliable further classification of these patients according to the number or timing of individual relapses after omalizumab withdrawal.

### 2.4. Clinical and Laboratory Assessments

Demographic, clinical, and laboratory data obtained before omalizumab initiation were retrospectively retrieved and compared between groups. Patients with unknown atopic status were included in the overall cohort description but excluded from the between-group comparison of atopic status.

Clinical variables included age at urticaria onset, sex, baseline disease activity, concomitant angioedema, history of drug allergy, atopic status, autoimmune thyroid disease, and previous systemic corticosteroid use for CSU exacerbations. Baseline disease activity was assessed using UAS7 values documented before omalizumab initiation. UAS7 ranges from 0 to 42 and comprises urticaria-free (0), well-controlled (1–6), mild (7–15), moderate (16–27), and severe (28–42) disease activity categories [10]. For the present analysis, the first three categories were combined, resulting in three study-specific categories: lower activity (0–15), moderate activity (16–27), and severe activity (28–42).

Baseline laboratory parameters included platelet-to-lymphocyte ratio (PLR), neutrophil-to-lymphocyte ratio (NLR), absolute eosinophil and basophil counts, total immunoglobulin E (IgE), anti-thyroid peroxidase antibody (anti-TPO), anti-TPO/total IgE ratio, antinuclear antibody (ANA), anti-double-stranded DNA antibody (anti-dsDNA), erythrocyte sedimentation rate (ESR), C-reactive protein (CRP), vitamin B12, and ferritin.

For categorical analyses, predefined cut-off values were used for total IgE (≥40 vs. <40 IU/mL) and anti-TPO (≥34 vs. <34 kU/L), based on previously reported thresholds [11], while CRP elevation was defined as >5 mg/L based on previous CSU research [12]. These categorizations were used only for descriptive and between-group comparisons. Vitamin B12 was analyzed as a continuous variable in both the between-group comparison and multivariable logistic regression.

### 2.5. Statistical Analysis

All analyses were conducted with IBM SPSS Statistics for Windows, version 21.0 (IBM Corp., Armonk, NY, USA). Continuous data are reported as mean ± standard deviation (SD) or median and interquartile range (IQR), depending on their distribution, whereas categorical data are summarized as numbers and percentages. Categorical variables were compared using Pearson’s chi-square test or Fisher’s exact test, as applicable, and continuous variables using the Mann–Whitney U test. Statistical significance was assessed using a two-sided threshold of *p* < 0.05.

Multivariable logistic regression was performed to evaluate factors independently associated with repeated omalizumab re-initiation. Given the limited sample size, a parsimonious model included age at urticaria onset, sex, baseline disease activity, and vitamin B12 level. Vitamin B12 was included as an exploratory variable because it was the only laboratory parameter showing a significant between-group difference in univariable analysis. Baseline disease activity was entered as an ordinal variable (1 = lower, 2 = moderate, and 3 = severe activity), and vitamin B12 as a continuous variable. Results were expressed as odds ratios (ORs) with 95% confidence intervals (CIs). The number of variables was limited to reduce the risk of overfitting. Because of the retrospective design and fixed number of eligible patients, a priori sample size calculation was not performed.

### 2.6. Declaration of AI-Assisted Language Editing

During the preparation of this manuscript, the authors used ChatGPT (GPT-5.6 Sol; OpenAI, San Francisco, CA, USA) for English-language editing and to improve sentence clarity. The authors reviewed and edited all AI-assisted text and take full responsibility for the content of this publication.

## 3. Results

A total of 46 patients with CSU were included in the study. The mean age at urticaria onset was 38.7 ± 12.5 years, and 27 patients (58.7%) were female. At baseline, 17 patients (37.0%) had lower disease activity (UAS7, 0–15), 14 (30.4%) had moderate disease activity (UAS7, 16–27), and 15 (32.6%) had severe disease activity (UAS7, 28–42). During the 12-month observation period following the first omalizumab withdrawal, 29 patients (63.0%) met the criteria for repeated omalizumab re-initiation, whereas 17 (37.0%) constituted the comparison group. Concomitant angioedema was present in 14 patients (30.4%), autoimmune thyroid disease in 3 (6.5%), and previous systemic corticosteroid use for CSU exacerbations in 24 (52.2%). Most patients were non-atopic (69.6%), and 71.7% had no documented history of drug allergy (Table 1).

Baseline demographic and clinical characteristics of the two groups are presented in Table 2. Median age at urticaria onset was 38 years (IQR, 31–46) in the repeated-treatment group and 35 years (IQR, 28–50) in the comparison group (*p* = 0.802), while the corresponding median intervals from urticaria onset to omalizumab initiation were 18 months (IQR, 6–36) and 10 months (IQR, 4–36), respectively (*p* = 0.501). Sex distribution, baseline UAS7 disease activity, angioedema, autoimmune thyroid disease, history of drug allergy, atopic status, ANA or anti-dsDNA positivity, and previous systemic corticosteroid use did not differ significantly between the groups (all *p* > 0.05). Categorical comparisons of total IgE, anti-TPO, and CRP also yielded no statistically significant group differences (all *p* > 0.05).

Among continuous laboratory parameters, median vitamin B12 levels were lower in the repeated-treatment group than in the comparison group [259 pg/mL (IQR, 209–317) vs. 335 pg/mL (IQR, 255–397), respectively; *p* = 0.034] (Table 3). None of the other continuous laboratory parameters, including PLR, NLR, absolute eosinophil and basophil counts, total IgE, anti-TPO, anti-TPO/total IgE ratio, ESR, CRP, and ferritin, differed significantly between the groups (all *p* > 0.05).

In multivariable logistic regression analysis, none of the included variables were independently associated with repeated omalizumab re-initiation (Table 4). The corresponding estimates were as follows: age at urticaria onset, OR 1.011 (95% CI, 0.955–1.070; *p* = 0.713); female sex, OR 0.861 (95% CI, 0.227–3.268; *p* = 0.826); baseline UAS7 disease activity per one-category increase, OR 0.707 (95% CI, 0.324–1.542; *p* = 0.384); and vitamin B12 level per 1-pg/mL increase, OR 0.995 (95% CI, 0.989–1.000; *p* = 0.066).

## 4. Discussion

In our cohort, 63.0% of patients experienced relapse requiring omalizumab re-initiation within 12 weeks of withdrawal in at least two treatment-withdrawal cycles during the 12-month observation period. Routinely available baseline demographic, clinical, and laboratory characteristics showed limited ability to distinguish these patients from the comparison group. Unlike studies primarily evaluating a single relapse after omalizumab withdrawal, the present study focused on recurrence across successive treatment-withdrawal cycles. This repeated relapse–retreatment pattern may be clinically relevant when planning long-term treatment and withdrawal strategies in CSU.

Relapse after omalizumab withdrawal is well recognized in CSU, although its frequency and timing vary considerably among studies [3,4,13,14]. In a large multicenter cohort of 470 patients, disease duration was associated with both first and second relapse after omalizumab withdrawal, whereas baseline UAS7 was associated with the first relapse only [13]. Other real-world studies have similarly documented frequent recurrence after withdrawal and restoration of disease control following omalizumab re-initiation [3,4,14]. More recently, a history of previous post-withdrawal relapse was independently associated with the risk of another relapse [5]. These findings support the clinical relevance of evaluating recurrence across more than one treatment-withdrawal cycle.

Evidence specifically addressing recurrence across successive omalizumab treatment cycles is limited. Kucharczyk et al. reported progressively shorter times to subsequent relapse across successive cycles, with nearly half of relapses occurring within the first six weeks after withdrawal; a greater number of previous relapses was also associated with subsequent recurrence [6]. Taken together, these observations suggest that the clinical course after omalizumab withdrawal may evolve across successive treatment cycles. Our study extends this perspective by focusing on patients requiring omalizumab re-initiation within 12 weeks of withdrawal in at least two treatment-withdrawal cycles, rather than on individual relapse episodes alone.

Studies investigating predictors of relapse after omalizumab withdrawal have yielded heterogeneous results. In a real-world cohort undergoing gradual omalizumab withdrawal, unsuccessful withdrawal was associated with longer disease duration and higher baseline total IgE and eosinophil counts [15]. In contrast, Koc Yildirim et al. reported that several conventional clinical parameters, total IgE, and CRP were not associated with recurrence, whereas anti-TPO and mean platelet volume emerged as potential markers [16]. Similarly, Ceravalls et al. reported associations of baseline CRP, total IgE, and treatment duration with sustained remission after optimized omalizumab withdrawal [17]. Together with the absence of independent associations for total IgE and anti-TPO reported by Sağun et al. [5], these findings illustrate the variability of proposed predictors across populations and withdrawal strategies. In our cohort, baseline disease activity, angioedema, atopic status, autoimmune thyroid disease, systemic corticosteroid use, total IgE, anti-TPO, CRP, eosinophil and basophil counts, and inflammatory indices did not significantly differ between groups. Overall, associations between routinely assessed baseline parameters and post-withdrawal outcomes have been inconsistent across studies; in our cohort, these parameters did not clearly distinguish patients who developed repeated relapse–retreatment cycles.

Importantly, post-withdrawal relapse should be distinguished from the clinical response to omalizumab. Omalizumab effectively suppresses the clinical manifestations of CSU but is not considered a disease-modifying treatment; therefore, a good clinical response during treatment does not necessarily imply that the underlying disease activity has resolved. After treatment withdrawal, persistent disease activity may become clinically apparent as recurrence of symptoms. This concept is supported by previous observations of relapse after treatment discontinuation even among patients who had achieved a prompt and complete response to omalizumab [9]. Consequently, post-withdrawal relapse may reflect underlying disease activity that is not adequately captured by currently available routine clinical or laboratory measures.

Vitamin B12 was the only laboratory parameter that differed significantly between groups in univariable analysis; however, this association was not retained after multivariable adjustment. Given the number of laboratory parameters examined and the absence of adjustment for multiple comparisons, the observed univariable difference may represent a chance finding. Therefore, the vitamin B12 finding should be regarded as exploratory and hypothesis-generating rather than as evidence of a predictive biomarker.

Several limitations should be considered when interpreting our findings. The retrospective, single-center design and modest sample size limit the generalizability of the results and reduce the statistical power of the analyses. In particular, the limited number of patients meeting the study outcome relative to the number of variables included in the multivariable model resulted in imprecise estimates, as reflected by the wide confidence intervals for some predictors. Accordingly, the regression findings should be interpreted cautiously, and the absence of statistically significant independent associations does not exclude a potential role for the evaluated factors in repeated relapse–retreatment cycles. In addition, decisions regarding omalizumab withdrawal and re-initiation were made as part of routine clinical practice rather than according to a prespecified protocol, which may have introduced variability in treatment management. The comparison group was defined by the absence of the prespecified repeated relapse–retreatment outcome and may therefore have encompassed different clinical courses after omalizumab withdrawal. Because the retrospective records did not allow these trajectories to be reliably characterized according to the number and timing of individual relapses, more detailed comparisons between specific relapse patterns could not be performed. More broadly, reliance on routinely collected clinical records may have resulted in incomplete or non-standardized assessments. Larger prospective, multicenter studies using standardized treatment withdrawal and follow-up protocols are needed to confirm these findings and to better characterize distinct patterns of recurrence after omalizumab withdrawal.

From a clinical perspective, repeated complete withdrawal may not represent the optimal long-term strategy for every patient once a recurrent relapse–retreatment pattern becomes apparent. Current recommendations emphasize individualized treatment de-escalation while acknowledging uncertainty regarding the optimal timing and method of omalizumab withdrawal [1,2,18]. Observational data suggest that gradual dose reduction or extension of dosing intervals may be associated with lower relapse rates than direct withdrawal, although comparative evidence remains limited and does not establish the superiority of a specific strategy [19,20]. Accordingly, individualized step-down approaches warrant prospective evaluation in patients experiencing repeated relapse–retreatment cycles.

In conclusion, repeated relapse requiring omalizumab re-initiation within 12 weeks after withdrawal in at least two treatment-withdrawal cycles was observed in a substantial proportion of patients with CSU. Routinely available baseline demographic, clinical, and laboratory parameters did not reliably distinguish these patients from the comparison group, highlighting the difficulty of identifying this pattern using conventional baseline assessments. These findings support further prospective investigation of individualized withdrawal and step-down strategies in patients who develop recurrent relapse–retreatment cycles.

## Figures and Tables

**Table 1:** Baseline Demographic and Clinical Characteristics of the Study Population.

Characteristic	Overall (n = 46)
Age at urticaria onset, years	38.7 ± 12.5
Sex	
Female	27 (58.7)
Male	19 (41.3)
Baseline UAS7 disease activity category	
Lower activity (0–15)	17 (37.0)
Moderate activity (16–27)	14 (30.4)
Severe activity (28–42)	15 (32.6)
Repeated omalizumab re-initiation during the 12-month observation period	
Yes	29 (63.0)
No	17 (37.0)
Concomitant angioedema	
Yes	14 (30.4)
No	32 (69.6)
Autoimmune thyroid disease	
Yes	3 (6.5)
No	43 (93.5)
History of drug allergy	
None	33 (71.7)
NSAIDs	7 (15.2)
β-lactam antibiotics	5 (10.9)
Multiple drug allergy	1 (2.2)
Atopic status	
Non-atopic	32 (69.6)
Perennial	8 (17.4)
Seasonal	1 (2.2)
Mixed	1 (2.2)
Unknown	4 (8.7)
ANA positivity	6 (13.0)
Anti-dsDNA positivity	1 (2.2)
Previous systemic corticosteroid use	
Yes	24 (52.2)
No	22 (47.8)

Data are presented as n (%) unless otherwise indicated. Age is presented as mean ± standard deviation. ANA, antinuclear antibody; NSAIDs, nonsteroidal anti-inflammatory drugs; UAS7, Urticaria Activity Score over 7 days.

**Table 2:** Baseline Clinical and Categorical Laboratory Characteristics by Treatment Group.

Characteristic	Comparison Group (n = 17)	Repeated-Treatment Group (n = 29)	*p* Value
Age at urticaria onset, years	35 (28–50)	38 (31–46)	0.802
Time from urticaria onset to omalizumab initiation, months	10 (4–36)	18 (6–36)	0.501
Sex			0.989
Female	10 (58.8)	17 (58.6)	
Male	7 (41.2)	12 (41.4)	
Baseline UAS7 disease activity category			0.597
Lower activity (0–15)	5 (29.4)	12 (41.4)	
Moderate activity (16–27)	5 (29.4)	9 (31.0)	
Severe activity (28–42)	7 (41.2)	8 (27.6)	
Concomitant angioedema			0.583
Yes	6 (35.3)	8 (27.6)	
No	11 (64.7)	21 (72.4)	
Autoimmune thyroid disease			0.545
Yes	2 (11.8)	1 (3.4)	
No	15 (88.2)	28 (96.6)	
History of drug allergy			0.739
Yes	4 (23.5)	9 (31.0)	
No	13 (76.5)	20 (69.0)	
Atopic status			0.715
Atopic	3 (18.8)	7 (26.9)	
Non-atopic	13 (81.2)	19 (73.1)	
Unknown	1 (5.9)	3 (10.3)	
Previous systemic corticosteroid use			0.936
Yes	9 (52.9)	15 (51.7)	
No	8 (47.1)	14 (48.3)	
ANA positivity			0.390
Positive	1 (5.9)	5 (17.2)	
Negative	16 (94.1)	24 (82.8)	
Anti-dsDNA positivity			1.000
Positive	0 (0.0)	1 (3.4)	
Negative	17 (100.0)	28 (96.6)	
Total IgE			0.655
≥40 IU/mL	14 (82.4)	26 (89.7)	
<40 IU/mL	3 (17.6)	3 (10.3)	
Anti-TPO			1.000
≥34 kU/L	4 (23.5)	6 (20.7)	
<34 kU/L	13 (76.5)	23 (79.3)	
CRP			0.359
>5 mg/L	10 (58.8)	13 (44.8)	
≤5 mg/L	7 (41.2)	16 (55.2)	

Continuous variables are presented as median (25th–75th percentile); categorical variables as n (%) within each treatment group. Mann–Whitney U test was used for continuous variables. Pearson’s χ^2^ test or Fisher’s exact test was used for categorical variables, as appropriate. Atopic-status percentages and the corresponding *p* value were calculated among patients with known atopic status (comparison n = 16; repeated-treatment n = 26); unknown status is shown separately. CRP elevation was defined as >5 mg/L.

**Table 3:** Baseline Continuous Laboratory Parameters by Treatment Group.

Laboratory parameter	Comparison Group (n = 17)	Repeated-Treatment Group (n = 29)	*p* Value
Platelet-to-lymphocyte ratio	148.1 (122.3–179.0)	139.5 (119.8–182.9)	0.554
Neutrophil-to-lymphocyte ratio	2.65 (2.15–4.51)	2.29 (1.84–2.79)	0.078
Absolute eosinophil count, cells/µL	108 (70–180)	130 (110–180)	0.561
Absolute basophil count, cells/µL	20 (10–30)	30 (20–45)	0.239
Total IgE, IU/mL	121.0 (67.1–246.0)	207.6 (138.0–484.0)	0.237
Anti-TPO, kU/L	1.75 (0.43–10.86)	2.08 (0.43–28.00)	0.682
Anti-TPO/total IgE ratio	0.012 (0.002–0.169)	0.006 (0.002–0.154)	0.946
ESR, mm/h	16.0 (7.0–23.0)	14.0 (8.0–25.0)	0.776
CRP, mg/L	7.40 (3.59–10.00)	4.30 (2.76–11.50)	0.649
Vitamin B12, pg/mL	335.0 (255.0–397.0)	259.0 (209.0–317.0)	0.034
Ferritin, ng/mL	30.7 (17.8–79.6)	63.3 (24.1–95.6)	0.339

Values are presented as median (25th–75th percentile). Between-group comparisons were performed using the Mann–Whitney U test. Anti-TPO, anti-thyroid peroxidase antibody; CRP, C-reactive protein; ESR, erythrocyte sedimentation rate; IgE, immunoglobulin E.

**Table 4:** Multivariable logistic regression analysis of factors associated with repeated omalizumab re-initiation.

Variable	Category/Unit	β Coefficient	Standard Error	OR	95% CI	*p* Value
Age at urticaria onset	Per 1-year increase	0.011	0.029	1.011	0.955–1.070	0.713
Sex	Female vs. male	−0.150	0.681	0.861	0.227–3.268	0.826
Baseline UAS7 disease activity	Per one-category increase	−0.347	0.398	0.707	0.324–1.542	0.384
Vitamin B12	Per 1-pg/mL increase	−0.005	0.003	0.995	0.989–1.000	0.066

Outcome: Repeated omalizumab re-initiation (yes = 1). Baseline UAS7 disease activity was entered as an ordinal variable (1 = lower activity, 2 = moderate activity, 3 = severe activity). Sex was coded as female vs. male. OR, odds ratio; CI, confidence interval; UAS7, Urticaria Activity Score over 7 days.

## Data Availability

The data that support the findings of this study are available from the corresponding author upon reasonable request.

## References

[1] Zuberbier T., Abdul Latiff A.H., Abuzakouk M., Aquilina S., Asero R., Baker D., Ballmer-Weber B., Bangert C., Ben-Shoshan M., Bernstein J.A. (2022). The international EAACI/GA^2^LEN/EuroGuiDerm/APAAACI guideline for the definition, classification, diagnosis, and management of urticaria. Allergy.

[2] Türk M., Yılmaz İ., Şahiner Ü.M., Kocatürk E., Şekerel B.E., Zuberbier T., Maurer M. (2022). Experience-based advice on stepping up and stepping down the therapeutic management of chronic spontaneous urticaria: Where is the guidance?. Allergy.

[3] Yang, Yu R., Qian W., Zheng Q., Xiong J., Chen S., Chen A., Chen J., Fang S., Huang K. (2023). Analysis of the Efficacy and Recurrence of Omalizumab Use in the Treatment of Chronic Spontaneous Urticaria and Chronic Inducible Urticaria. Int. Arch. Allergy Immunol..

[4] Ornek S., Kocaturk E. (2021). A patient-oriented approach to long-term use of omalizumab in chronic spontaneous urticaria. Cutan. Ocul. Toxicol..

[5] Sağun F., Çölkesen F., Gerek M.E., Kolak S., Harman E., Savaş Ş.A., Yiğitdöl İ., Ergün Ü.Y., Arslan Ş. (2025). Clinical and Laboratory Predictors of Relapse within One Year after Omalizumab Discontinuation in Chronic Spontaneous Urticaria: A Real-Life Cohort Study. Int. Arch. Allergy Immunol..

[6] Kucharczyk A., Marczyk K., Kucharczyk B., Plisko R., Perkowska J., Owczarek W., Jahnz-Różyk K. (2024). Predicting relapse in chronic spontaneous urticaria: A retrospective cohort study evaluating omalizumab withdrawal regimens. Allergy.

[7] Sussman G., Hébert J., Gulliver W., Lynde C., Yang W.H., Papp K., Gooderham M., Chambenoit O., Khalil S., DeTakacsy F. (2020). Omalizumab Re-Treatment and Step-Up in Patients with Chronic Spontaneous Urticaria: OPTIMA Trial. J. Allergy Clin. Immunol. Pract..

[8] Chen Y., Yu M., Huang X., Tu P., Shi P., Maurer M., Zhao Z. (2021). Omalizumab treatment and outcomes in Chinese patients with chronic spontaneous urticaria, chronic inducible urticaria, or both. World Allergy Organ. J..

[9] Asero R. (2018). Efficacy of omalizumab 150 mg/month as a maintenance dose in patients with severe chronic spontaneous urticaria showing a prompt and complete response to the drug. Allergy.

[10] Stull D., McBride D., Tian H., Arnau A.G., Maurer M., Marsland A., Balp M.-M., Khalil S., Grattan C. (2017). Analysis of disease activity categories in chronic spontaneous/idiopathic urticaria. Br. J. Dermatol..

[11] Larenas-Linnemann D. (2023). Biomarkers of autoimmune chronic spontaneous urticaria. Curr. Allergy Asthma Rep..

[12] Özçeker D., Can P.K., Terzi Ö., Ornek S.A., Değirmentepe E.N., Kızıltac K., Sarac E., Kocatürk E. (2023). Differences between adult and pediatric chronic spontaneous urticaria from a cohort of 751 patients: Clinical features, associated conditions and indicators of treatment response. Pediatr. Allergy Immunol..

[13] Marzano A.V., Genovese G., Casazza G., Fierro M.T., Dapavo P., Crimi N., Ferrucci S., Pepe P., Liberati S., Pigatto P.D. (2019). Predictors of response to omalizumab and relapse in chronic spontaneous urticaria: A study of 470 patients. J. Eur. Acad. Dermatol. Venereol..

[14] Metz M., Ohanyan T., Church M.K., Maurer M. (2014). Retreatment with omalizumab results in rapid remission in chronic spontaneous and inducible urticaria. JAMA Dermatol..

[15] Maoz-Segal R., Levenberg G., Levy T., Haj-Yahia S., Shavit R., Machnes-Maayan D., Lifshitz-Tunitsky Y., Niznik S., Offengenden I., Iancovich-Kidon M. (2024). Omalizumab withdrawal outcomes in chronic spontaneous urticaria are linked with baseline IgE and eosinophil levels. World Allergy Organ. J..

[16] Koc Yildirim S., Erbagci E., Demirel Ogut N., Unal S., Gokyayla E. (2026). Predictors of relapse after omalizumab withdrawal in chronic spontaneous urticaria: Role of baseline autoimmunity and hematologic indices. Allergy Asthma Proc..

[17] Ceravalls J., Giménez-Arnau A.M., Expósito-Serrano V., Chico N.F., Moya A.L., Bielsa I., Ribó P., Mascaró-Hereza B., Bonfill-Ortí M., Spertino J. (2025). Redefining omalizumab discontinuation in chronic spontaneous urticaria: The value of optimization and predictive factors of relapse. A 52-week multicenter study. Actas Dermosifiliogr..

[18] Sánchez J., Pite H., Gómez R.M., Ansotegui I.J., Canonica G.W., Dávila I., Ferrer M., Abujeta J.L.G., Martin B., Morais-Almeida M. (2025). Chronic spontaneous urticaria remission definition and therapy stepping down: World Allergy Organization position paper. J. Allergy Clin. Immunol..

[19] Üstün P., Adışen E. (2025). Factors influencing relapse after omalizumab in chronic urticaria. Does the method of discontinuation influence relapse?. Dermatol. Pract. Concept..

[20] Salman A., Aktas M., Apti Sengun O. (2021). Remission of chronic spontaneous urticaria following omalizumab with gradually extended dosing intervals: Real-life data. Australas. J. Dermatol..

